
JOURNAL INFORMATION
==============================
NLM Title Abbreviation: EURASIP J Bioinform Syst Biol
Journal ID: EURASIP J Bioinform Syst Biol
NLM Journal ID: 101263720

EURASIP Journal on Bioinformatics and Systems Biology

ISSN: 1687-4145
EISSN: 1687-4153
Publisher: Springer

ARTICLE INFORMATION
==============================
PMCID: PMC3171329
PMID: 17728840
DOI: 10.1155/2007/17321
Article ID: 1687-4153-2007-17321
Article version: 1
Subjects: Editorial

Genetic Regulatory Networks

Dougherty Edward R 1 2 edward@ece.tamu.edu
Akutsu Tatsuya 3 takutsu@kuicr.kyoto-u.ac.jp
Dan Cristea Paul 4 pcristea@dsp.pub.ro
Tewfik Ahmed H 5 tewfik@umn.edu
1 Department of Electrical & Computer Engineering, College of Engineering, Texas A&M University, College Station, TX 77843-3128, USA
2 Translation Genomics Research Institute, Phoenix, AZ 85004, USA
3 Bioinformatics Center, Institute for Chemical Research, Kyoto University, Gokasho, Uji, Kyoto 611-0011, Japan
4 Digital Signal Processing Laboratory, Department of Electrical Engineering, "Politechnica" University of Bucharest, Bucharest 060032, Romania
5 Department of Electrical and Computer Engineering, Institute of Technology, University of Minnesota, Minneapolis, MN 55455, USA
Publication date: 2007
Electronic publication date: 2007 Aug 2
Volume: 2007
Issue: 1
First page: 17321
Last page: 17321
Received 2007 Jun 3; Accepted 2007 Jun 3
Copyright: Copyright © 2007 Edward R. Dougherty et al.
Copyright year: 2007
Copyright holder: Edward R. Dougherty et al.
License: This is an open access article distributed under the Creative Commons Attribution License, which permits unrestricted use, distribution, and reproduction in any medium, provided the original work is properly cited.
License URL: https://creativecommons.org/licenses/by/4.0/

==============================

Hindawi Publishing Corporation
EURASIP Journal on Bioinformatics and Systems Biology
Volume 2007, Article ID 17321, 2 pages
doi:10.1155/2007/17321

*Editorial*

### Genetic Regulatory Networks

**Edward R. Dougherty,[1,2] Tatsuya Akutsu,[3] Paul Dan Cristea,[4] and Ahmed H. Tewfik[5]**

[1] *Department of Electrical & Computer Engineering, College of Engineering, Texas A&M University, College Station, TX 77843-3128, USA*
[2] *Translation Genomics Research Institute, Phoenix, AZ 85004, USA*
[3] *Bioinformatics Center, Institute for Chemical Research, Kyoto University, Gokasho, Uji, Kyoto 611-0011, Japan*
[4] *Digital Signal Processing Laboratory, Department of Electrical Engineering, "Politechnica" University of Bucharest, 060032 Bucharest, Romania*
[5] *Department of Electrical and Computer Engineering, Institute of Technology, University of Minnesota, Minneapolis, MN 55455, USA*

Received 3 June 2007; Accepted 3 June 2007

Copyright © 2007 Edward R. Dougherty et al. This is an open access article distributed under the Creative Commons Attribution License, which permits unrestricted use, distribution, and reproduction in any medium, provided the original work is properly cited.

Systems biology aims to understand the manner in which the parts of an organism interact in complex networks, and systems medicine aims at basing diagnosis and treatment on a systems level understanding of molecular interaction, both intra-and inter-cellular. Ultimately, the enterprise rests on characterizing the interaction of the macromolecules constituting cellular machinery. Genomics, a key driver in this enterprise, involves the study of large sets of genes and proteins, with the goal of understanding systems, not simply components. The major goal of translational genomics is to characterize genetic regulation, and its effects on cellular behavior and function, thereby leading to a functional understanding of disease and the development of systems-based medical solutions.

To achieve this goal it is necessary to develop nonlinear dynamical models that adequately represent genomic regulation and to develop mathematically grounded diagnostic and therapeutic tools based on these models. Signals generated by the genome must be processed to characterize their regulatory effects and their relationship to changes at both the genotypic and phenotypic levels. Owing to the complex regulatory activity within the cell, a full understanding of regulation would involve characterizing signals at both the transcriptional (RNA) and translational (protein) levels; however, owing to the tight connection between the levels, a goodly portion of the information is available at the transcriptional level, and owing to the availability of transcription-based microarray technologies, most current studies utilize mRNA expression measurements. Since transcriptional (and posttranscriptional) regulation involves the processing of numerous and different kinds of signals, mathematical and computational methods are required to model the multivariate influences on decision-making in genetic networks.

Construction of a network model is only the beginning of biological analysis. Understanding a gene network means understanding its dynamics, especially its long-run behavior. For instance, it has been conjectured that the stationary distribution characterizes phenotype. It is in terms of dynamics that issues such as stability, robustness, and therapeutic effects must be examined. Indeed, it seems virtually impossible to design targeted treatment regimens that address a patient's individual regulatory structure without taking into account the stochastic dynamics of cell regulation. From the perspective of systems medicine, perhaps the most important issue to be addressed is the design of treatment policies based on the external control of regulatory network models, since this is the route to the design of optimal therapies, both in terms of achieving desired changes and avoiding deleterious side effects.

As a discipline, signal processing involves the construction of model mathematical systems, including systems of differential equations, graphical networks, stochastic functional relations, and simulation models. And if we view signal processing in the wide sense, to include estimation, classification, automatic control, information theory, networks, and coding, we see that genomic signal processing will play a central role in the development of systems medicine. There is a host of important and difficult problems, ranging over issues such as inference, complexity reduction, and the control of high-dimensional systems. These represent an exciting challenge for the signal processing community and a chance for the community to play a leading role in the future of medicine.

*Edward R. Dougherty*
*Tatsuya Akutsu*
*Paul Dan Cristea*
*Ahmed H. Tewfik*
